# BioInfograph: An Online Tool to Design and Display Multi-Panel Scientific Figure Interactively

**DOI:** 10.3389/fgene.2021.784531

**Published:** 2022-01-05

**Authors:** Kejie Li, Jessica Hurt, Christopher D. Whelan, Ravi Challa, Dongdong Lin, Baohong Zhang

**Affiliations:** Translational Biology, Biogen Inc., Cambridge, MA, United States

**Keywords:** bioinformatics, infographic, high-resolution, Scalable Vector Graphics, multi-panel figure

## Abstract

Many fit-for-purpose bioinformatics tools generate plots to interpret complex biological data and illustrate findings. However, assembling individual plots in different formats from various sources into one high-resolution figure in the desired layout requires mastery of commercial tools or even programming skills. In addition, it is a time-consuming and sometimes frustrating process even for a computationally savvy scientist who frequently takes a trial-and-error iterative approach to get satisfactory results. To address the challenge, we developed bioInfograph, a web-based tool that allows users to interactively arrange high-resolution images in diversified formats, mainly Scalable Vector Graphics (SVG), to produce one multi-panel publication-quality composite figure in both PDF and HTML formats in a user-friendly manner, requiring no programming skills. It solves stylesheet conflicts of coexisting SVG plots, integrates a rich-text editor, and allows creative design by providing advanced functionalities like image transparency, controlled vertical stacking of plots, versatile image formats, and layout templates. To highlight, the sharable interactive HTML output with zoom-in function is a unique feature not seen in any other similar tools. In the end, we make the online tool publicly available at https://baohongz.github.io/bioInfograph while releasing the source code at https://github.com/baohongz/bioInfograph under MIT open-source license.

## Introduction

Popular computational biology databases such as Reactome (Jassal et al., 2020), WikiPathways (Martens et al., 2021), and visualization tools such as Coral (Metz et al., 2018) and ComplexHeatmap (Gu et al., 2016) often produce biological images in Scalable Vector Graphics (SVG) format. SVG is an Extensible Markup Language (XML)-based vector image format, scalable to any resolution without blurry pixelization that happens in other popular image formats such as png, gif, and jpg. This format has become one of the most broadly used image outputs adopted by many data analysis tools used by computational biologists, notably R (Venables et al., 2002), ggplot2 (Wickham, 2016), and numerous R and Bioconductor (Gentleman et al., 2004) packages. In addition, SVG is usually set as the default image output by many JavaScript-based plotting libraries like D3 (Bostock et al., 2011). To point out, these SVG images are rendered naturally by modern web browsers including Chrome, Firefox, Safari, and Microsoft Edge.

**FIGURE 1:**
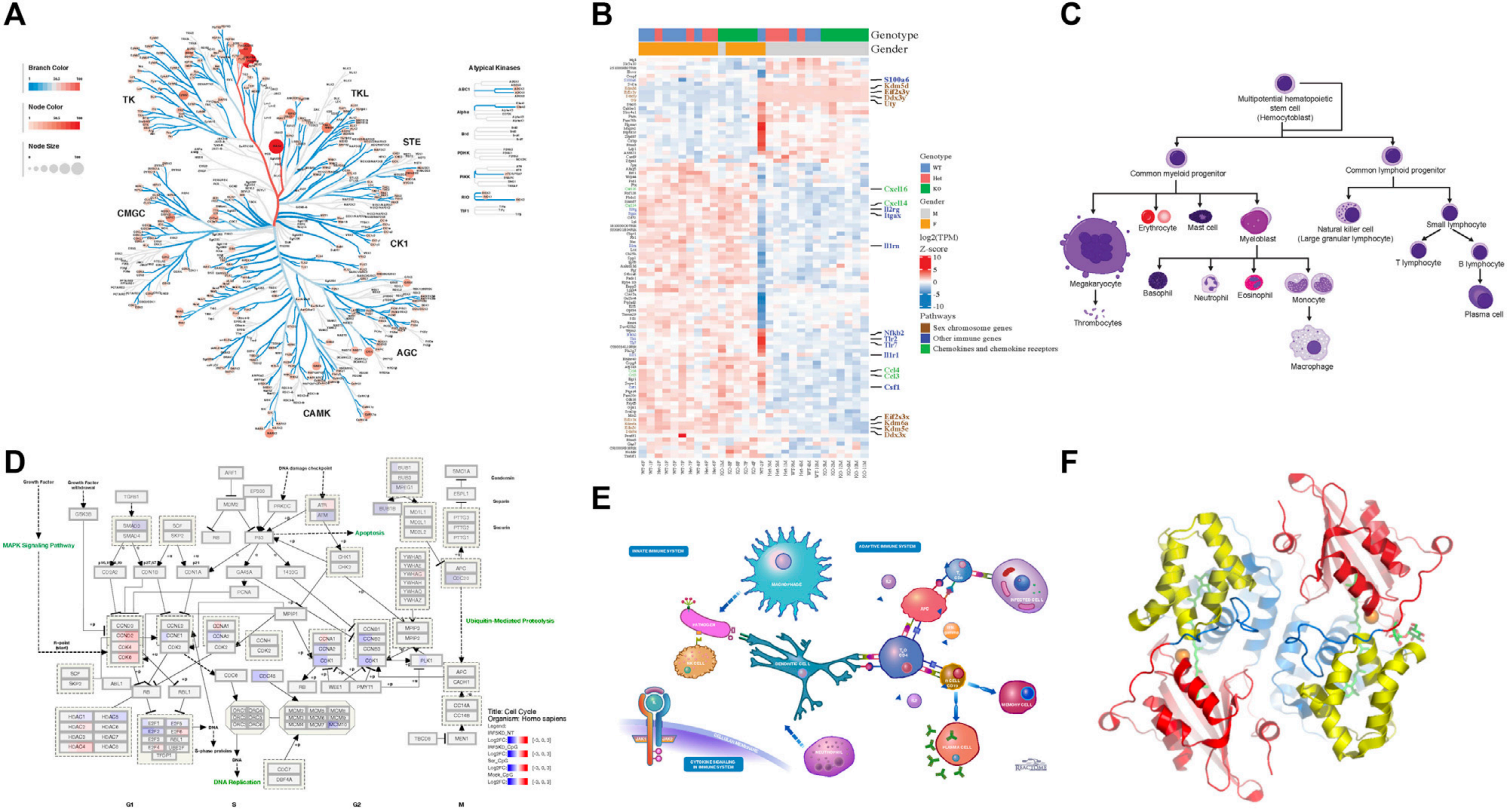
High-resolution plots generated by various tools are arranged by the online tool, bioInfograph, to produce a composite plot. Unless specified, the source plot is in Scalable Vector Graphics (SVG) format. **(A)** Human kinome tree generated by Coral web app. **(B)** Gene expression heatmap by R package ComplexHeatmap. **(C)** Blood cell lineage from Wikimedia Commons (https://bit.ly/2Wjc5aS). **(D)** Human cell cycle pathway diagram from WikiPathways. **(E)** Human immune system illustration from Reactome. **(F)** Protein 3D structure ribbon form in png format by PyMOL (DeLano, 2000). An interactive version of the figure for the enlarged view of individual panels is available at https://bit.ly/39ClQnD.

**FIGURE 2:**
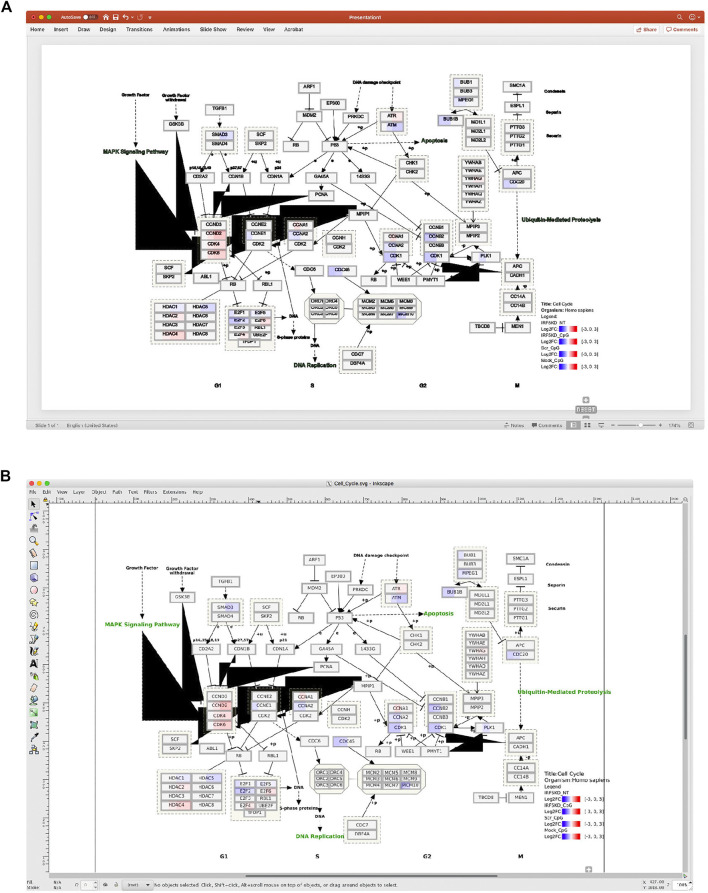
An example of a pathway diagram from WikiPathways is not properly rendered by **(A)** Microsoft PowerPoint or **(B)** Inkscape. Please note the unexpected black triangles generated by both tools and loss of green color in the text (e.g., DNA replication) by PowerPoint, while the same Scalable Vector Graphics (SVG) image is rendered perfectly by bioInfograph as shown in Figure 1D.

Composing multi-panel publication-ready figures, such as the one presented in Figure 1, usually poses a challenge for biologists with no or modest programming skills after gathering individual plots from various sources in diversified formats, such as png, gif, jpg, tiff, pdf, and svg. Nevertheless, creating graphical abstracts like Figure 1 to give a high-level comprehensive story becomes a routine task in biological publication. And often, such illustration is required to be in high resolution. Biologists usually turn to user-friendly commercial tools, such as Microsoft PowerPoint, as viable options to arrange such plots. But these tools either cannot deal with complex pathway diagrams in SVG format from WikiPathways, or render this format in low resolution with missing colors, sometimes even in malformed appearance as shown in Figure 2.

A previously developed web-based plot designing tool, canvasDesigner (Zhang et al., 2018), attempted to provide a solution but with limited success. It fails to handle stylesheet conflicts caused by SVG files from different tools and lacks flexibility in design where images are required to overlay onto each other. Moreover, singular input image format and rudimentary text support hinder its usability. To address these major shortcomings, we revamped the new version to accept more image formats in bioInfograph beyond only SVG, as acquiring such format might be unfeasible in certain circumstances such as scanned gel images, and we improved usability tremendously by implementing advanced functions outlined in the *Materials and Methods* section.

## Materials and Methods

### Implementation and Usage of BioInfograph

**FIGURE 3:**
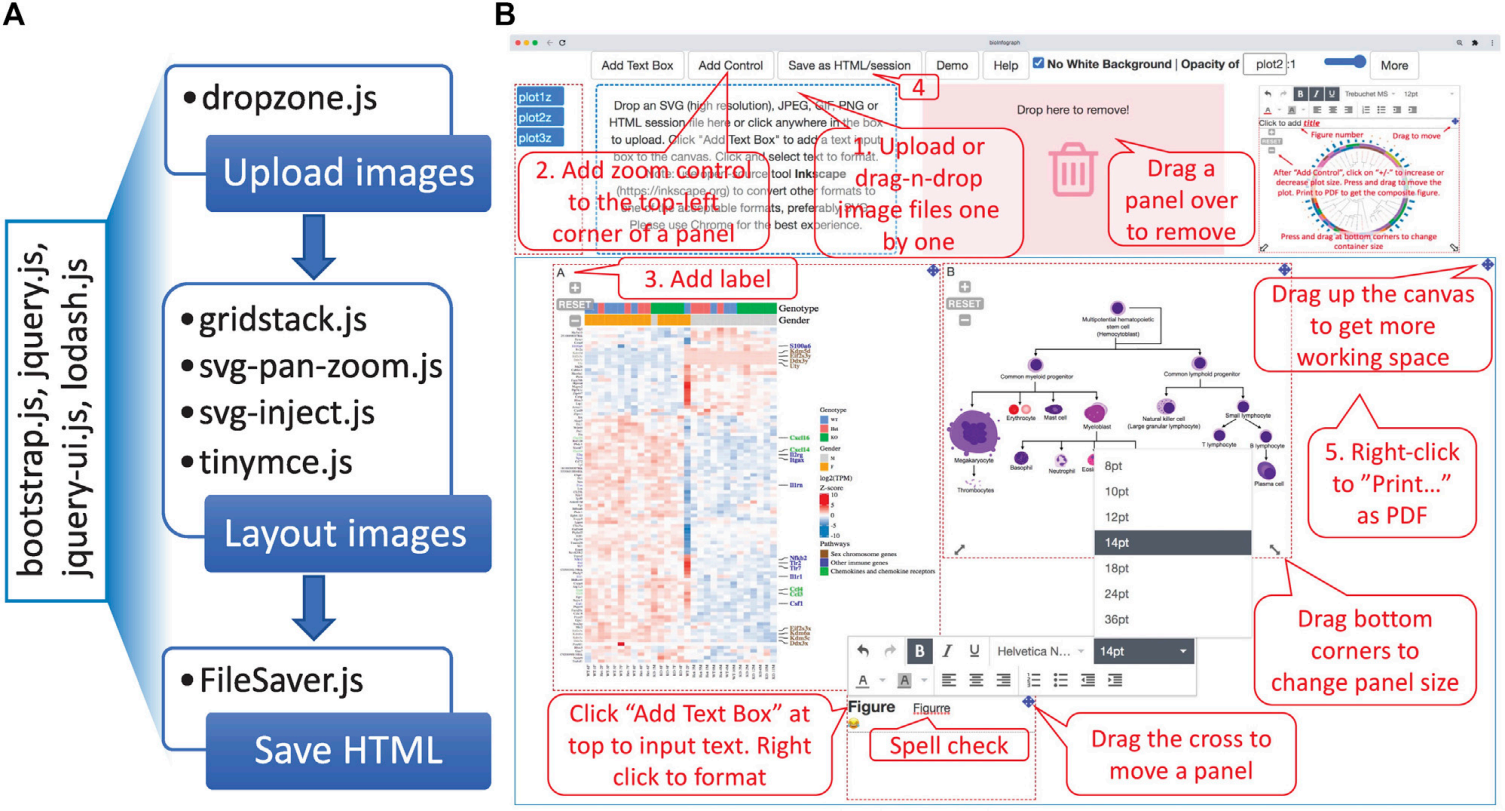
Overview of the software architecture and main features. **(A)** The one-page web app is powered by general JavaScript libraries listed in the rotated box and specific libraries used in each of three functional modules, “Upload images,” “Layout images,” and “Save HTML.” **(B)** BioInfograph allows users to easily arrange multiple plots in Scalable Vector Graphics (SVG), png, jpeg, or gif format exported by other tools. Each plot can be adjusted in size and placed freely on the canvas. A minimal workflow is outlined by numbered callout boxes in red, while important features not required by the minimal workflow are briefed in un-numbered boxes.

With simplicity and accessibility in mind, it is implemented as a one-page, client-only, web-based application without the server-side component, available online at https://baohongz.github.io/bioInfograph. Written in plain JavaScript language, bioInfograph takes advantage of open-source JavaScript libraries including common ones like jQuery, bootstrap, and lodash. As shown in Figure 3A, other special JavaScript libraries are listed under each of three functional modules, “Upload images,” “Layout images,” and “Save HTML,” to show the design of the software. First, dropzone.js makes it easy to upload or drag and drop image files to the tool. The content of uploaded or dropped files will be put on the canvas for layout. The source code in the library is modified to allow emitting “previewReady” status when an image is fully loaded into memory and displayed in the preview box; see https://bit.ly/3Gup4Zp for details. Second, gridstack.js is used to layout draggable, resizable, responsive bootstrap-friendly panels in a grid on the designing canvas. Each panel in the grid holds one image that can be panned or zoomed in and out by attached control provided by svg-pan-zoom.js. Modifications are made in gridstack.js to preserve inline styles, including positions, size, and z-index in order to drag a panel to an accurate location instead of pre-defined stops; see https://bit.ly/3CaOg3T for details. Functions of tinymce.js and svg-inject.js libraries are discussed in the following related sections. Third, FileSaver.js is utilized to save image content and associated metadata about size, position, opacity, and zoom scale in an HTML file. When taken together, an intuitive user interface is built and shown in action as illustrated in Figure 3B, where control elements are located at the top, functional modules in the middle, and a movable, dynamically resizable canvas at the bottom. A very basic workflow is outlined by numbered callout boxes consisting of five steps: 1) uploading images; 2) adding pan-zoom control to fine-tune image size and position; 3) adding labels; 4) saving the work as an HTML file; and 5) printing as PDF. While not required in the minimal setting, all other un-numbered boxes highlight important features to smooth the design process, such as moving the canvas up to create more working space, changing the size of an individual panel, dropping a panel to a trash bin, and adding text box for typing paragraphs of text with spell checking. Due to the space limitation of the figure, some features are discussed in more detail below.

Besides online access, users can install it as a desktop app by downloading the html page or creating a shortcut of the page on the desktop by following the instruction in GitHub repo, https://bit.ly/3wTxoxk. To be aware, the tool is fully tested in the Chrome browser, which provides the best experience.

### Flexible Text Input

**FIGURE 4:**
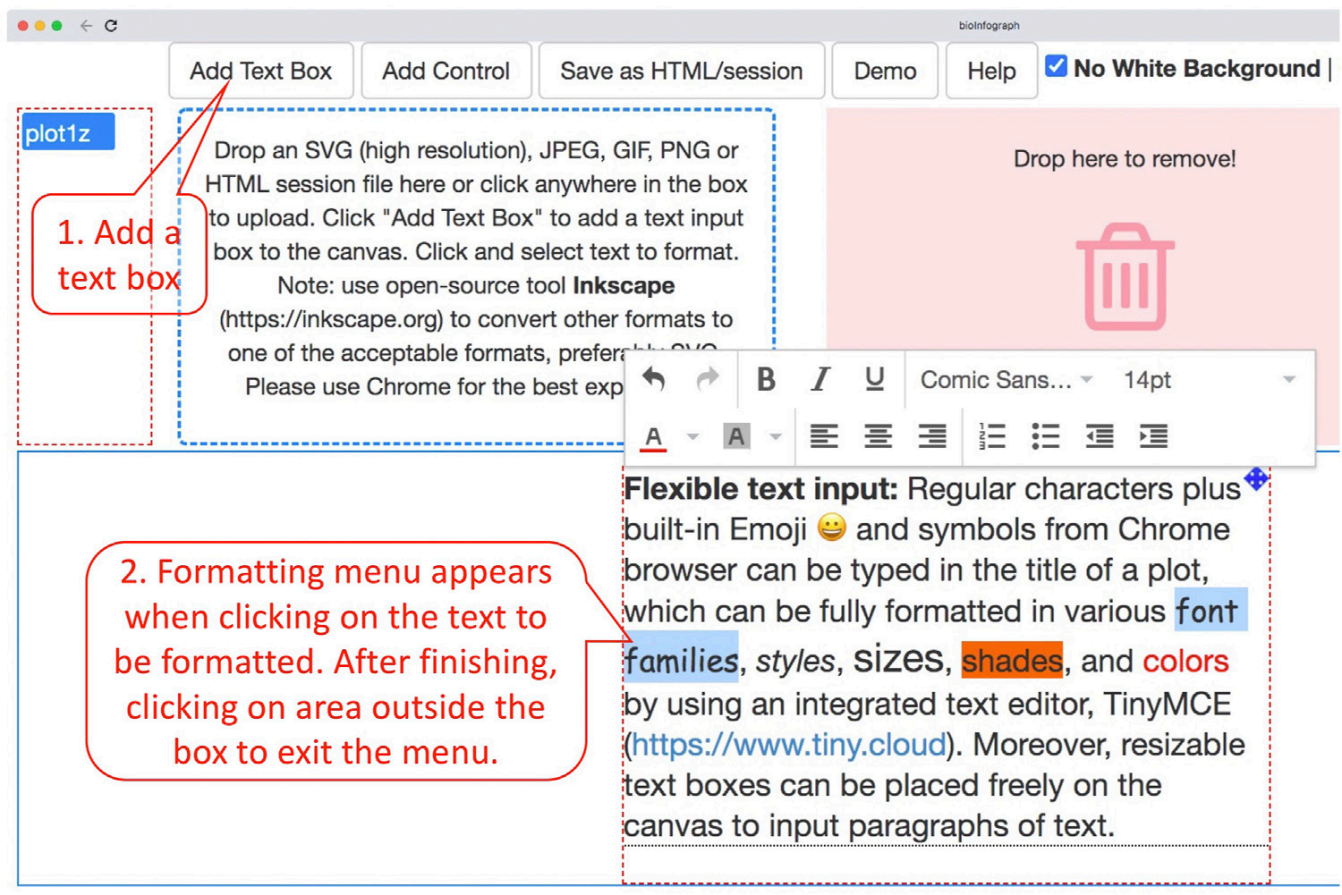
Input of paragraphs of text by clicking on “Add Text Box” and then typing in the resizable box that was added to the canvas a moment ago. Clicking on the text will fire up the formatting menu, while clicking on any area outside the box will exit the menu. Formatting is always applied to selected text.

Regular characters plus built-in Emoji and symbols from Chrome browser can be typed in the title of a plot, which can be fully formatted in various font families, styles, sizes, shades, and colors by using an integrated text editor, TinyMCE (https://www.tiny.cloud). Moreover, resizable text boxes can be placed freely on the canvas to input paragraphs of text by following the instructions in Figure 4. The markdown language has gained popularity in authoring simple documents especially within R and GitHub communities. A very simple markdown processor is enabled by using tinymce’s text pattern plugin that matches the following patterns (source code block from index.html) in the text and applies corresponding formats on these patterns; e.g., “*test*” will become “*test*” in the editor.

textpattern_patterns: [

{start: '*', end: '*', format: 'italic'},

{start: '**', end: '**', format: 'bold'},

{start: '#', format: 'h1'},

{start: '##', format: 'h2'},

{start: '###', format: 'h3'},

{start: '####', format: 'h4'},

{start: '#####', format: 'h5'},

{start: '######', format: 'h6'},

{start: '1. ', cmd: 'InsertOrderedList'},

{start: '* ', cmd: 'InsertUnorderedList'},

{start: '- ', cmd: 'InsertUnorderedList'}

],

### Versatile Image Formats

Besides the SVG format, bioInfograph accepts directly additional popular image formats including png, gif, and jpg as input. For other formats like tiff or pdf, free tools such as Inkscape (https://inkscape.org) (Bah, 2007) or pdf2svg (https://bit.ly/2NVtj6E) can be utilized to convert these to one of the acceptable formats, preferably SVG.

### Stylesheet Conflict

Since stylesheet definitions in SVG files are always applied globally to style elements, they share the same parse tree when multiple inline SVGs are embedded in a single document. Therefore, style overwriting and component id collisions can occur and upset the rendering in canvasDesigner as shown in Supplementary Figure S1. To overcome these shortcomings, bioInfograph automatically converts global definitions into inline styles embedded in each targeting element individually, stores it locally, and then removes these definitions from the global scope to solve the overwriting issue. Then, it utilizes a modified version of svg-inject.js (see https://bit.ly/3Gus3kz for details) to make ids in the document unique by appending original ids with a suffix in the form of “--inject-X”, where X is a running number that is incremented with each added SVG image.

### Vertical Stacking

**FIGURE 5:**
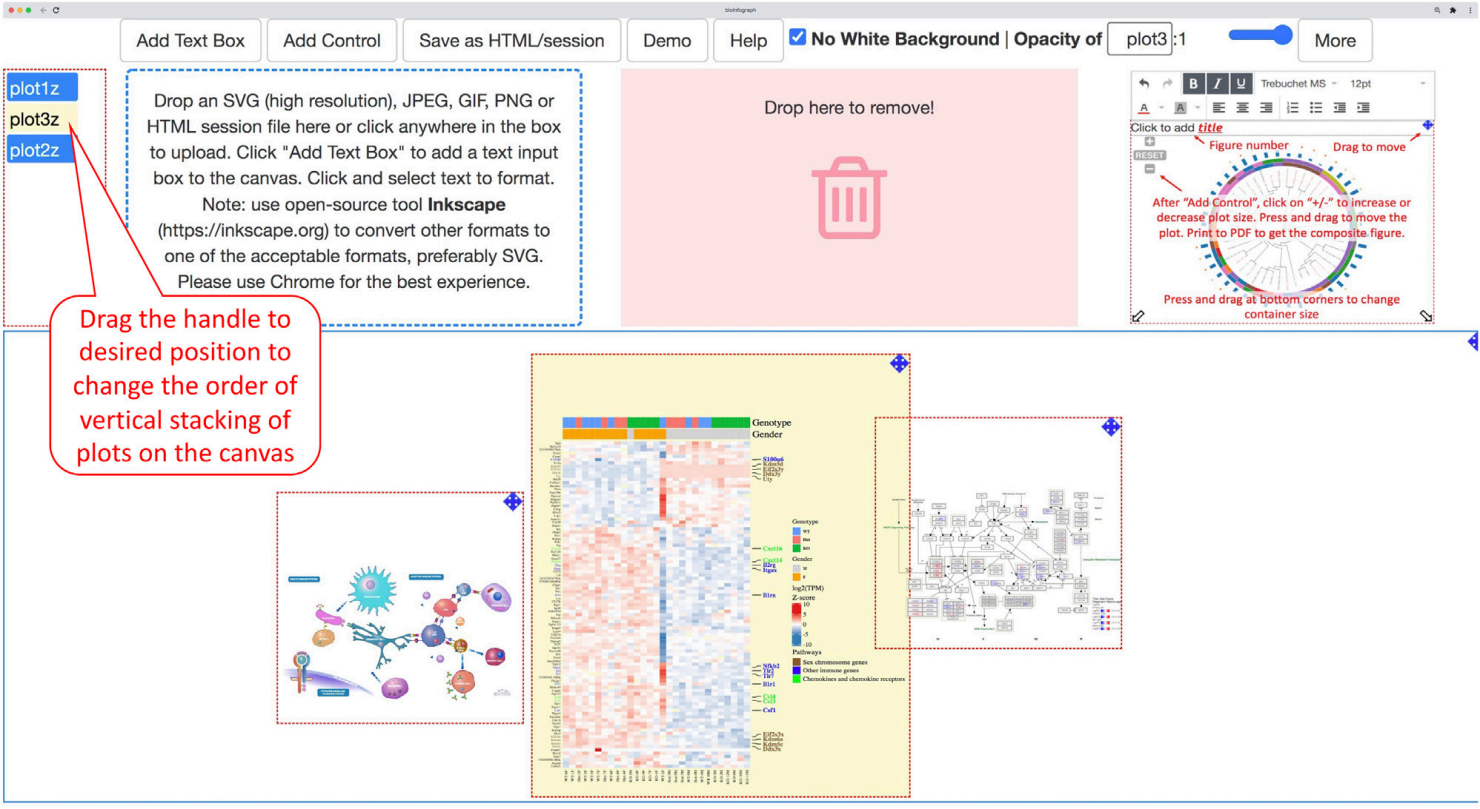
Each plot will get a button handle when it is loaded onto the canvas. A plot and the linked handle will be highlighted in yellow when hovering the mouse over a handle. Dragging the handle and dropping it at the desired position among these buttons will change the relative vertical stacking, also known as z-index of the plot. The bottom position represents the top layer of the stack of plots on the canvas.

Each image is associated with a vertically stacked control button. Desired vertical stacking order (z-index) is attainable by moving these control buttons up or down by mouse as demonstrated in Figure 5, which provides an additional dimension for creative design that often requires overlapped images in a certain order.

### Image Transparency

**FIGURE 6:**
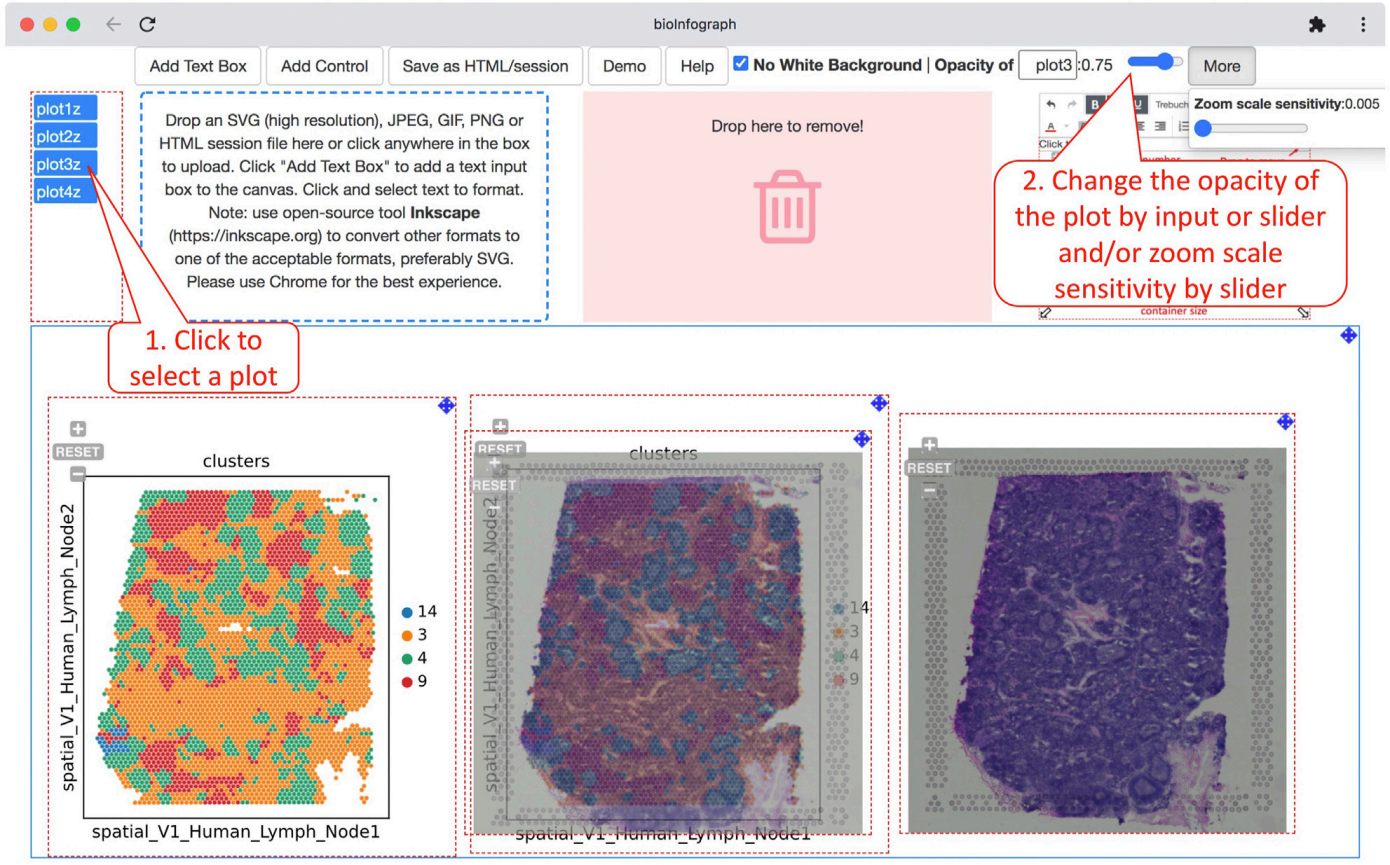
Overlaying colored spatial clustering plot and histological image to illustrate the relationship of histological features and clusters based on 10X Genomics spatial transcriptomics data (https://bit.ly/3F0xKWD). Users can adjust the opacity of a selected plot by clicking a plot handle to select the plot and then typing a number or using the slide to change the value. The zoom scale sensitivity of the plot is tuned to the smallest number for fine alignment of overlaid plots.

The white background in the SVG file is optionally removable to make it transparent so that plots can be overlaid onto each other to create appealing art. Opacities of individual images can be adjusted granularly as well to make a comprehensive effect of overlaid images as showcased in visualizing spatial transcriptomics data, which is displayed in Figure 6. In this use case, vertical stacking of gene expression data on top of histopathology images or vice versa with adjustable transparency is a crucial visualization capability to investigate the relationship between the transcriptional signals and disease pathology.

### Interactive HTML Output and Saved Session

**FIGURE 7:**
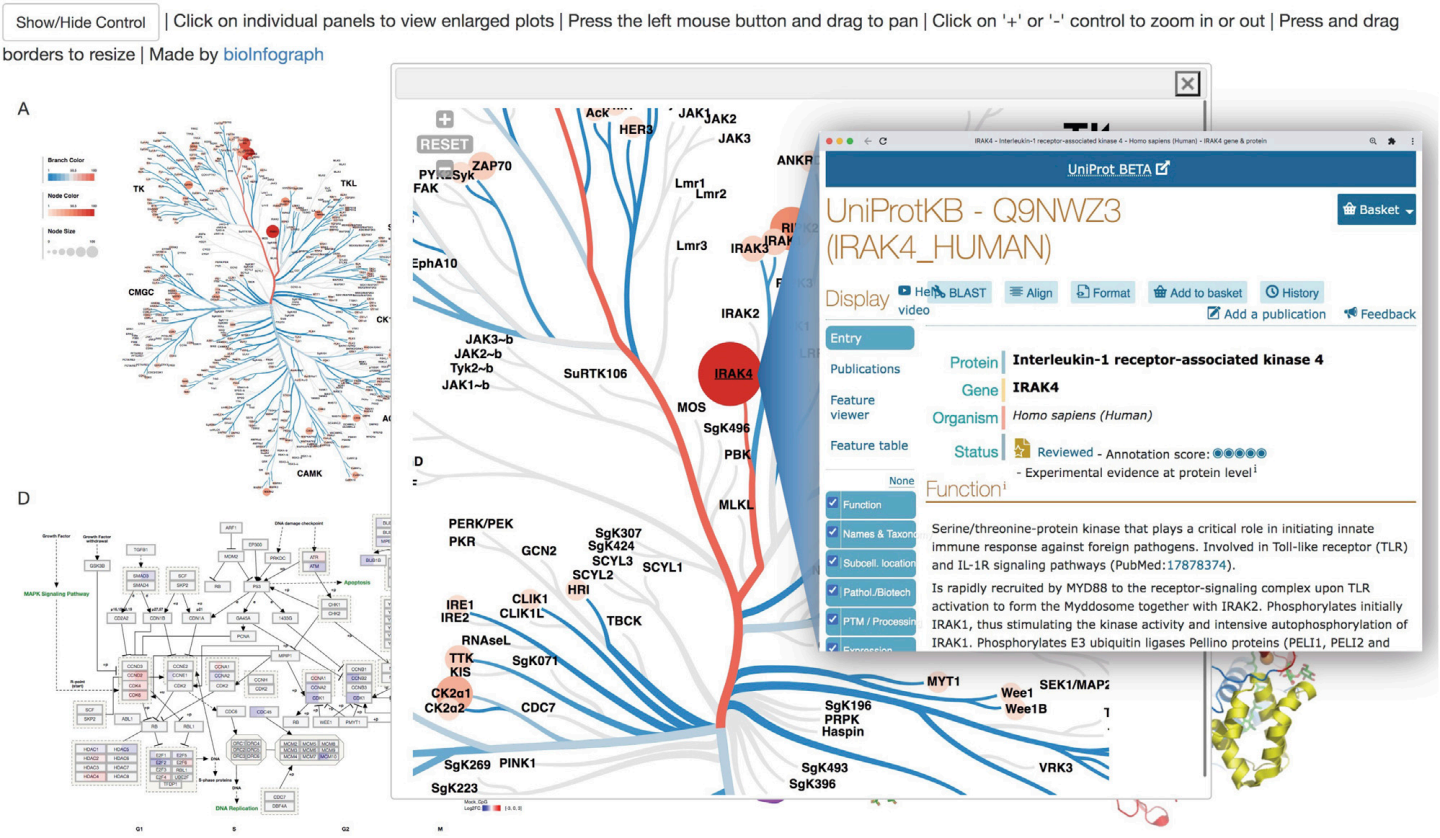
Interactive online HTML presentation of Figure 1 with zoom-in and link-out features. Clicking on an individual plot will bring up a popup window with the enlarged zoomable version. Links in Scalable Vector Graphics (SVG) are active, so clicking on “IRAK4” on the node in the phylogenetic tree will show detailed information about the protein in UniProt database.

The finished work can be saved as a self-contained HTML file with necessary JavaScript code embedded for easy sharing by email or hosting at GitHub-like services as exemplified at https://bit.ly/39ClQnD. An individual plot can be enlarged and further zoomed in to view details in high resolution by clicking on the plot and then the button with a plus sign in the popup window. Unique to this HTML presentation, links to detailed information of proteins in UniProt database (The UniProt Consortium, 2021) are active in panel A of the interactive figure as shown in Figure 7. Therefore, bioInfograph output can act as an information portal beyond mere pictures by embedding links to dissipated computational biology resources in SVG figures. Meanwhile, the saved HTML file also serves as a session file that can be loaded back into the tool to restore the work for further modification.

## Results

We developed bioInfograph, an interactive web-based tool with a focus on computational biology, which arranges high-resolution images in various formats, mainly SVG, to produce one multi-panel publication-quality composite figure in both PDF and interactive HTML formats in a user-friendly manner, requiring no programming skills.

We compared it with several popular tools to illustrate the advanced features of bioInfograph. Among the six tools listed in Table 1, except patchwork (Pedersen, 2019), which is a command line based tool, the rest offers an interactive user-friendly interface. In addition, bioInfograph and canvasDesigner are conveniently accessible web-based tools. Regarding image formats, Adobe Acrobat and patchwork will not take SVG as input natively, while PowerPoint and Inkscape have issues when rendering complex pathway diagrams in SVG format as shown in Figure 2. Although canvasDesigner and bioInfograph share many common features, bioInfograph breaks the limitations of canvasDesigner by solving conflicting stylesheet issues, accepting images in various formats, overlaying images in any order vertically, adjusting image transparency, and providing flexible text input. In summary, we outline a comparison scorecard of features among these tools including both open source solutions and popular commercial tools available to the authors in Table 1.

**TABLE 1:** Comparison scorecard of figure design tools.

	BioInfograph v1.0	Canvasdesigner v1.0	MS powerpoint v16.3	Adobe acrobat pro DC v2020.006	Patchwork v1.0	Inkscape v0.92
Open source/cost	Yes/free	Yes/free	No/license fee	No/license fee	Yes/free	Yes/free
Multi-image formats	Yes	No	Yes	Yes	No	Yes
Rendering speed	Fast	Fast	Fast	Fast	Fast	Slow
Text input	Yes	No	Yes	Yes	Yes	Yes
Interactive HTML output	Yes	Yes	No	No	No	No
SVG input	Yes	Yes	Yes	No	No	Yes
SVG stylesheet compatibility	Yes	No	Yes	N/A	N/A	No
Image transparency	Yes	No	Yes	No	N/A	Yes
Saving session	Yes	No	Yes	No	No	No
Installation free	Yes	Yes	No	No	No	No

SVG, Scalable Vector Graphics.

## Conclusion

BioInfograph is an open-source and publicly available web-based tool that can be accessed online or downloaded as a desktop application. It has the most feasible features to improve productivity in the case of creating high-resolution multi-panel figures for scientific publication. Furthermore, the innovative HTML output brings a new way of illustrating high-resolution figures interactively with unlimited zoom-in capability, which could be a nice feature for journals to incorporate in online publishing.

## Data Availability

The original contributions presented in the study are included in the article/Supplementary Material. Further inquiries can be directed to the corresponding author.
