# Supplementary material for: BioInfograph: An Online Tool to Design and Display Multi-Panel Scientific Figure Interactively
Source: Front Genet. 2022 Jan 5;12:784531. doi: 10.3389/fgene.2021.784531 (PMC8766334; doi:10.3389/fgene.2021.784531)
Supplement: Supplementary file 1 [file Image1.pdf]

Supplementary Material

1 Supplementary Figures

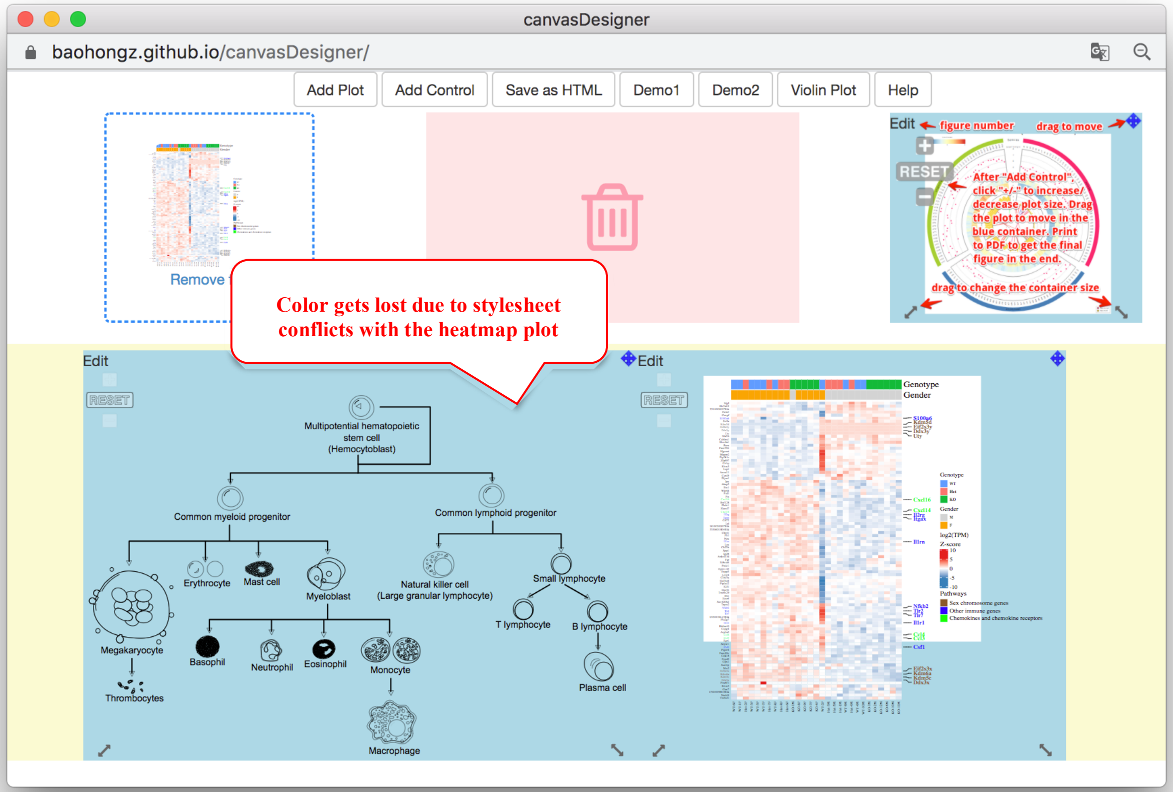

Supplementary Figure 1. Human blood cell lineage plot loses all of its colors due to conflicting stylesheet definition in heatmap plot by using canvasDesigner tool. On the contrary, localization of styles to individual elements in bioInfograph solves the issue as shown in Figure 1C.

2 Supplementary Links

Google Chrome (https://www.google.com/chrome) is recommended for optimal use of the web-based tool, bioInfograph.

bioInfograph: https://baohongz.github.io/bioInfograph

bioInfograph source code: https://github.com/baohongz/bioInfograph

Example SVG files: https://github.com/baohongz/bioInfograph/tree/gh-pages/SVG

Inkscape: Tool to edit SVG files, convert image files from other formats to SVG format. https://inkscape.org

pdf2svg: A simple PDF to SVG converter using the Poppler and Cairo libraries. https://github.com/dawbarton/pdf2svg

SVGOMG: SVG optimizer to minimize the size of SVG files. https://jakearchibald.github.io/svgomg

Coral: Clear and customizable visualization of human kinome data. http://phanstiel-lab.med.unc.edu/CORAL

ComplexHeatmap: Complex heatmaps are efficient to visualize associations between different sources of data sets and reveal potential patterns. https://www.bioconductor.org/packages/release/bioc/html/ComplexHeatmap.html

Wikimedia Commons: media file collection of 59.4 million freely usable media files to which anyone can contribute. https://commons.wikimedia.org/wiki/Main_Page

WikiPathways: a database of biological pathways maintained by and for the scientific community. https://www.wikipathways.org/index.php/WikiPathways

Reactome: a free, open-source, curated and peer-reviewed pathway database. Our goal is to provide intuitive bioinformatics tools for the visualization, interpretation and analysis of pathway knowledge to support basic research, genome analysis, modeling, systems biology and education. https://reactome.org

PyMOL: a user-sponsored molecular visualization system on an open-source foundation, maintained and distributed by Schrödinger. https://pymol.org
